# Dopa-Responsive Dystonia: Functional Analysis of Single Nucleotide Substitutions within the 5’ Untranslated *GCH1* Region

**DOI:** 10.1371/journal.pone.0076975

**Published:** 2013-10-04

**Authors:** Ioanna A. Armata, Leonora Balaj, John K. Kuster, Xuan Zhang, Shelun Tsai, Andreas A. Armatas, Trisha J. Multhaupt-Buell, Roy Soberman, Xandra O. Breakefield, Hiroshi Ichinose, Nutan Sharma

**Affiliations:** 1 Department of Neurogenetics, Massachusetts General Hospital and Program in Neuroscience, Harvard Medical School, Boston, Massachusetts, United States of America; 2 Department of Radiology, Massachusetts General Hospital and Program in Neuroscience, Harvard Medical School, Boston, Massachusetts, United States of America; 3 Department of Biology, University of Patras, Rio Patras, Greece; 4 Renal Unit, Department of Medicine, Massachusetts General Hospital, Boston, Massachusetts, United States of America; 5 Graduate School of Bioscience and Biotechnology, Tokyo Institute of Technology, Yokohama, Japan; National Institutes of Health, United States of America

## Abstract

**Background:**

Mutations in the *GCH1* gene are associated with childhood onset, dopa-responsive dystonia (DRD). Correct diagnosis of DRD is crucial, given the potential for complete recovery once treated with L-dopa. The majority of DRD associated mutations lie within the coding region of the *GCH1* gene, but three additional single nucleotide sequence substitutions have been reported within the 5’ untranslated (5’UTR) region of the mRNA. The biologic significance of these 5’UTR *GCH1* sequence substitutions has not been analyzed.

**Methodology/Principal Findings:**

Luciferase reporter assays, quantitative real time PCR and RNA decay assays, combined with bioinformatics, revealed a pathogenic 5’UTR *GCH1* substitution. The +142C>T single nucleotide 5’UTR substitution that segregates with affected status in DRD patients, substantially attenuates translation without altering RNA expression levels or stability. The +142C>T substitution disrupts translation most likely by creating an upstream initiation start codon (uAUG) and an upstream open reading frame (uORF).

**Conclusions/Significance:**

This is the first *GCH1* regulatory substitution reported to act at a post-transcriptional level, increasing the list of genetic diseases caused by abnormal translation and reaffirming the importance of investigating potential regulatory substitutions in genetic diseases.

## Introduction

Dopa-responsive dystonia (DRD) was first described by Segawa et al. [1]. It is a movement disorder of childhood onset, typically beginning in a foot followed by spread, over several years, to involve other limbs, rendering walking difficult. DRD displays a diurnal fluctuation with increasing severity over the course of the day and a marked, sustained response to low therapeutic doses of levodopa. However, diagnosis remains a challenge with many cases being misdiagnosed as cerebral palsy or spastic diplegia.

DRD is frequently caused by heterozygous mutations in the guanosine triphosphate (GTP) cyclohydrolase 1 gene (*GCH1*) [2,3], with this form of dystonia now referred to as DYT5 [4]. GTP cyclohydrolase 1 catalyzes the first step in the biosynthesis of (BH4). BH4 is a cofactor for three aromatic amino acid hydroxylases, including tyrosine hydroxylase (TH), which is the rate-limiting enzyme in dopamine biosynthesis [5]. Thus, low levels of BH4, due to mutations in the *GCH1* gene, can lead to a reduction in dopamine synthesis and dysfunction of dopaminergic nigrostriatal neurochemical transmission [6].

**Figure 1:**
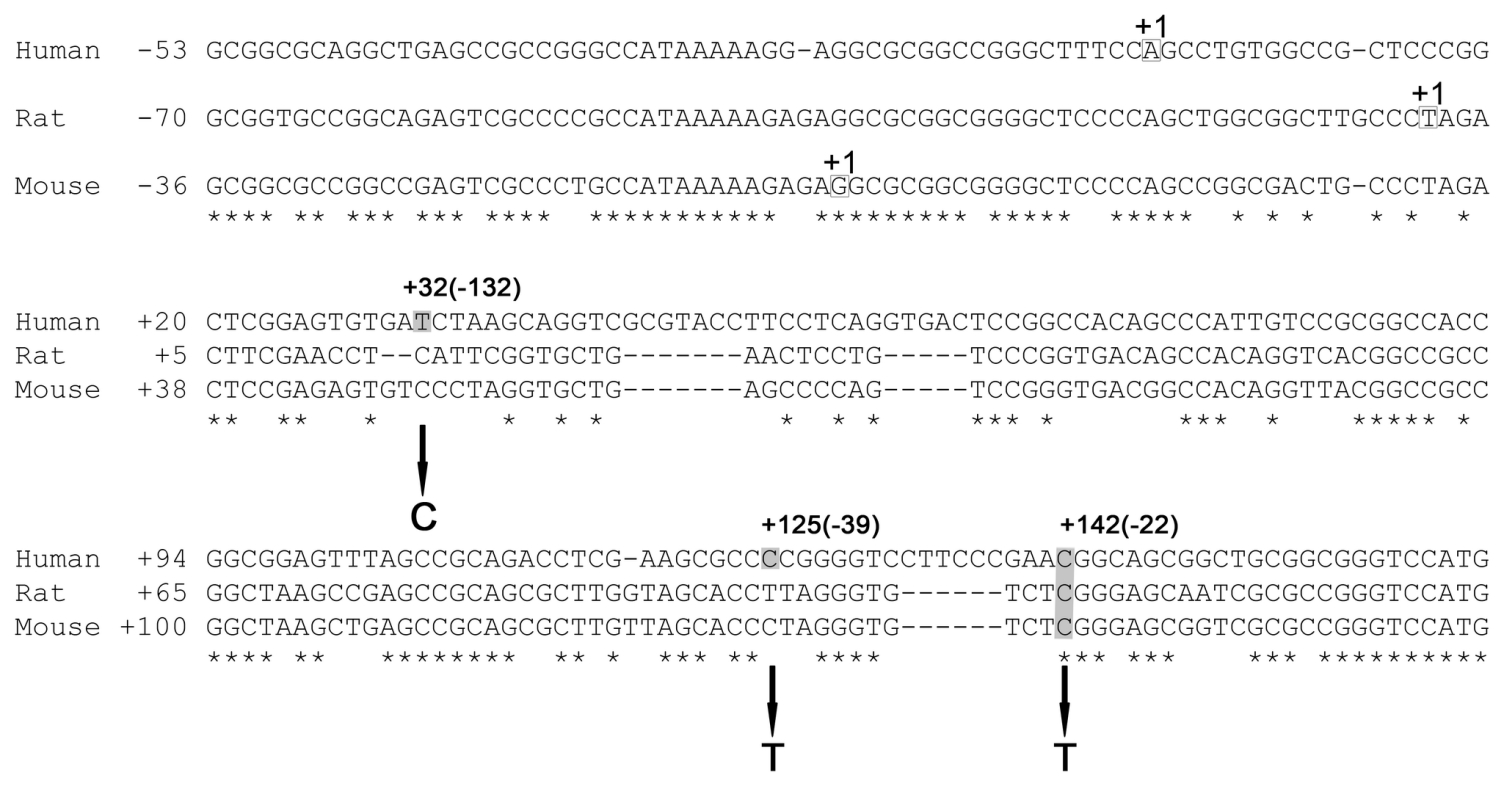
Alignment of the human, mouse and rat 5′ upstream *GCH1* regions. Nucleotides are numbered from the transcription start site (+1) as previously identified for the human (top), rat (middle) and mouse (bottom) *GCH1* genes. Position of each *GCH1* 5’UTR substitution identified to date is shaded and the nucleotide substitution is shown beneath the sequence. An asterisk (*) indicates sequence identity across the three species.

To date, 114 different mutations have been identified in the *GCH1* gene in patients with DRD (http://www.biopku.org/authorisation/login.asp) involving, in their majority, missense and nonsense mutations in all 6 six exons [2,3,7–11], as well as large or multiexonic deletions and exon frameshifts [12–15]. Noncoding *GCH1* mutations have also been reported spanning its 5’ upstream region [16–18] and intron-exon splice sites [19–21]. Within the 5’ upstream region of the human *GCH1* gene, three different single base pair (bp) substitutions have been identified at positions -22C>T [16,18], -39C>T and -132T>C [17], relative to the *GCH1* translation start codon (ATG), in DRD individuals who lack mutations in the coding region (Figure 1). As the *GCH1* transcription start site has been identified, we have now demarcated the substitutions relative to the *GCH1* transcription start site (+1) to be in accordance with revised genetic nomenclature. Thus, the -22C>T substitution is referred to as +142C>T, the -39C>T as +125C>T and the -132T>C as +32T>C. The +125C>T and +32T>C substitutions were identified as co-existing in *cis* in a single subject with DRD [17], making it impossible to independently determine hereditary disease association for each substitution. The +142C>T substitution has been identified in two different reports, one of a single individual with DRD [16] and another in multiple generations of a family with DRD, in whom the +142C>T substitution segregated with affected status [18]. Whether any of these single nucleotide substitutions have an impact on *GCH1* expression levels has not been investigated.

In the present paper, we demonstrated that among the three aforementioned 5’UTR *GCH1* substitutions, only the +142C>T is a functional substitution that decreases protein expression. However, neither mRNA level nor stability were affected by this substitution. The 5’UTR mediated regulation of translation may reflect alterations in secondary RNA structure, changes in RNA binding motifs (RBPs) and associated proteins, or the creation of upstream open reading frames (uORFs) [22–25]. The +142C>T substitution within the *GCH1* 5’UTR introduces an upstream AUG (uAUG) that potentially creates an out-of-frame uORF. This uORF partially overlaps with the physiological ORF (pORF) and likely acts in a competitive manner to down regulate translation. To our knowledge, this is the first report of a translational disruption causing DRD or any other type of dystonia.

## Methods

### Cell cultures

The human neuroblastoma cell line SK-N-BE(2)-M17 was purchased from ATCC (Manassas, VA) and maintained in MEM/F-HAM, supplemented with 10% fetal bovine serum and 1% penicillin/streptomycin, at 37°C and 5% CO_2_. Lymphocytes isolated from DRD patients and healthy individuals were Epstein Barr Virus (EBV) - transformed to stable cell lines, as previously described [26] and maintained in RPMI 1640 with 5% fetal calf serum, 1% penicillin/streptomycin and 2 mM glutamine at 37°C and 5% CO_2_.

### Patients and controls

Lymphoblastoid cultures were established from 10 family members, spanning three generations, in whom the +142C>T substitution segregated with affected status [18]. Six subjects were heterozygous for the +142 SNP (C/T) and met the clinical criteria for DRD [7], while the remaining four family members were homozygous for the +142C nucleotide (C/C) and had no evidence of dystonia or other basal ganglia dysfunction on examination. Two additional control samples (+142C/C) were utilized from the discarded sample collection of the Massachusetts General Hospital Neurogenetics DNA Diagnostic Laboratory. Full *GCH1* gene sequencing (exons 1-6), including intron/exon junctions was performed for all samples and no other nucleotide changes were found [18]. The local institutional review board approved the study. All participants gave informed consent.

### Reporter constructs and mutagenesis

**Figure 2:**
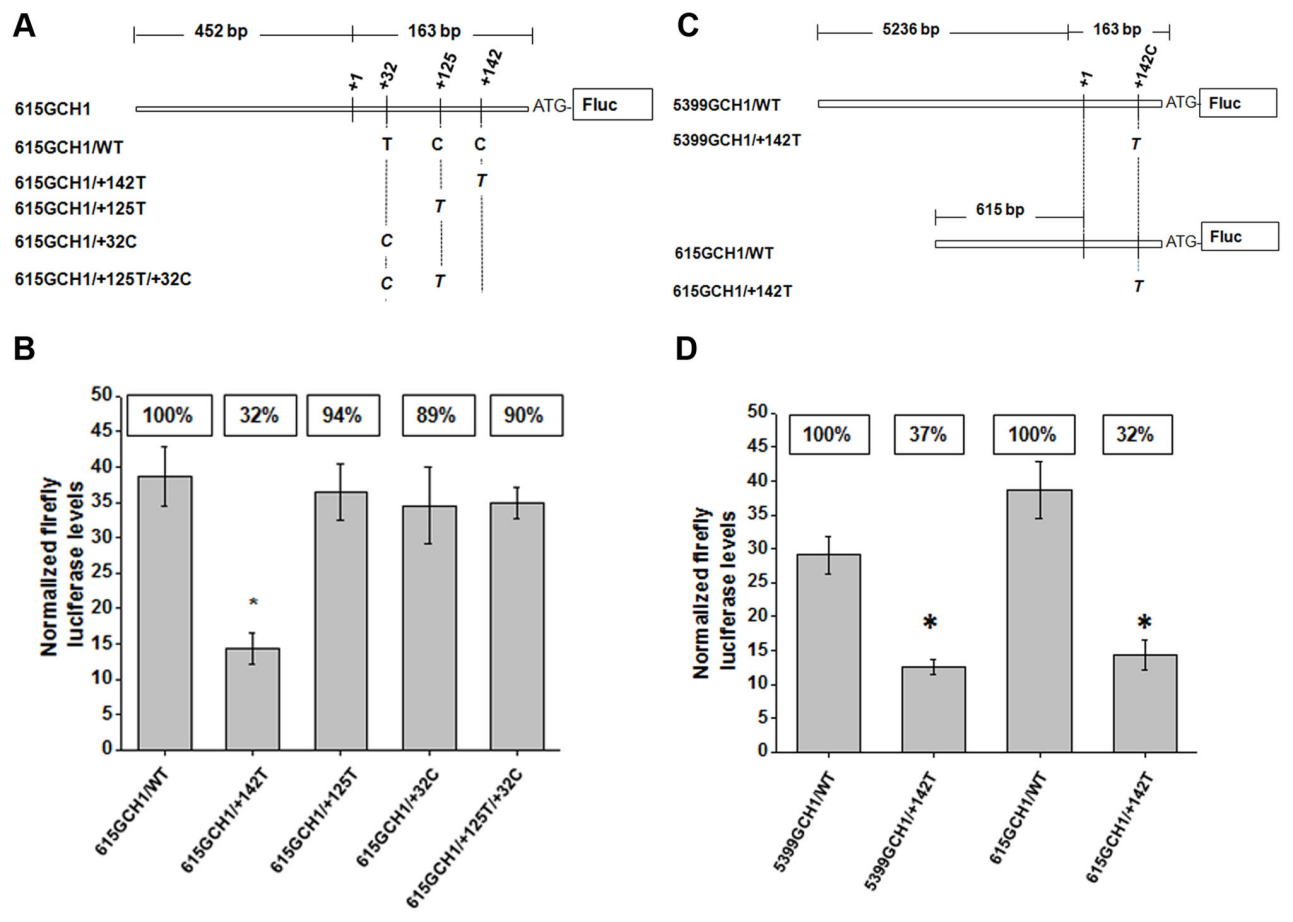
Effects of the +142C>T, the +125C>T and the +32T>C 5’UTR *GCH1* single nucleotide substitutions on luciferase activity. (**A**) Schematic representation of the luciferase reporter constructs bearing a 615 bp fragment of the 5’ upstream *GCH1* region followed by the luciferase gene (Fluc) with either the wild type sequence (+32T/+125C/+142C) or the identified substitutions (+142T, +125T, +32C or +32C/+125T). (**B**) SK-N-BE2-M17 cells were transiently transfected with each Fluc construct indicated in the bottom of each graph bar and the CMV-Rluc. Results are shown as the mean ± S.E.M. of relative Fluc normalized to Rluc activity. The % activity of each construct relative to the wild type (set as 100%) is indicated above each bar (n=4, Student’s t-test * = *P* ≤ 0.005). (**C**) Schematic representation of the reporter constructs bearing a 5399 or a 615 bp fragment of the 5’ upstream *GCH1* region followed by the Fluc with either a C (5399GCH1/WT and 615GCH1/WT) or a T (5399GCH1/+142T and 615GCH1/+142T) at +142. (**D** SK-N-BE2-M17 cells were transiently transfected with each Fluc construct indicated in the bottom of each graph bar and the CMV-Rluc. Results are shown as the mean ± S.E.M. of relative Fluc normalized to Rluc activity. The % activity of each construct relative to the wild type (set as 100%) is indicated above each bar (n=4, Student’s t-test * = *P* ≤ 0.005).

Human *GCH1* luciferase reporter constructs have been previously described [9]. We utilized a 615 bp and a 5399 bp 5’ upstream *GCH1* fragment linked to the firefly luciferase (Fluc) cDNA in the promoter-less basic Picca Gene Basic Vector 2 (PGV-B2) (Toyo Ink Co., Ltd, Tokyo) [27] referred to as 615GCH1/WT (wild type) and 5399GCH1/WT, respectively (Figure 2A & 2C). Mutant reporter constructs were generated from the parental 615GCH1/WT by replacing the +142C with a T (615GCH1/+142T), the +125C with a T (615GCH1/+125T), the +32T with a C (615GCH1/+32C), the +125C with a T and the +32T with a C in the same construct (615GCH1/+125T/+32C) (Figure 2A). A mutant reporter construct was generated from the parental 5399GCH1/WT by replacing the +142C with a T (5399GCH1/+142T) (Figure 2C). All mutant products were generated using the QuikChange site-directed mutagenesis kit (Stratagene, La Jolla, CA) and appropriate sets of primers (sequences available upon request). All constructs were confirmed by DNA sequencing.

### Transfections and luciferase assays

Transient transfections were performed using Lipofectamine 2000 (Invitrogen, Carlsbad, CA) in 1.0 to 1.5 x 10^6^ cells/well plated onto 24-well tissue culture plates. For each Fluc reporter construct used in transfections, two independent plasmid Maxi-preps (Qiagen, Valencia, CA) were used. Each transfection consisted of 0.8 μg DNA of Fluc reporter construct and 0.4 μg CMV-*Renilla* luciferase (Rluc) vector bearing Rluc under the cytomegalovirus (CMV) promoter, as an internal control of transfection efficiency (Promega, Madison, WI). Luciferase activity was analyzed 40 hrs post-transfection, using a dual luciferase reporter kit (Promega) and a luminometer (Dynex, Richfield, NM). Luciferase assays were repeated at least four times, and within each experiment each construct was transfected in quadruplicate. Results were expressed as a mean of quadruplicate measures (+ S.E.M.) of Fluc normalized to Rluc activity. Statistical significance was determined by Student’s t-test, comparing the activity of each mutant construct with the wild type parental construct.

### Quantitative real-time PCR

RNA was isolated from SK-N-BE(2)-M17 cells transfected with the 615GCH1/WT or the 615GCH1/+142T construct or from 5.0 x 10^6^ human lymphoblastoid cells using the miRNA purification kit (Qiagen) and then subjected to DNase treatment (Qiagen). Two hundred ng of RNA was then reverse transcribed using Omniscript (Qiagen) and qPCR reactions were performed in a 25 µl reaction mixture using Power SYBR® Green PCR Master Mix (Applied Biosystems, Foster City, CA) and 160 nM of each primer. Amplification conditions consisted of: (1) 1 cycle of 50°C, 2 min; (2) 1 cycle of 95°C, 10 min; (3) 40 cycles of 95°C, 15 sec; and 60°C, 1 min, and (4) 1 cycle of 95°C, 15 sec; 60°C, 20 sec; and 95°C, 15 sec, run on the 7000 ABI Prism PCR system (Applied Biosystems). Fluc cycle threshold (Ct) values were normalized to Rluc while the *GCH1* Ct values were normalized to the housekeeping gene hypoxanthine phosphoribosyltransferase 1 (*HPRT1*). Normalized Ct values were then converted to relative mRNA levels. All qRT-PCRs were repeated 4 times for each gene and each sample was done in triplicate. Primer dimers were excluded by evaluation of dissociation curve and agarose gel electrophoresis. Primer sequences were as follows: Rluc forward- GATAACTGGTCCGCAGTGGT and reverse – ACCAGATTTGCCTGATTTGC; Fluc forward- ATCCATCTTGCTCCAACACC and reverse – TTTTCCGTCATCGTCTTTCC; *GCH1* forward CCTACTCGTCCATCCTGAGC and reverse GGACCTTTCCAACAAATGGA; *HPRT1* forward TGACACTGGCAAAACAATGCA and reverse GGTCCTTTTCACCAGCAAAGCT. For mRNA decay assays, 24hrs post-transfection, SK-N-BE(2)-M17 cells were supplemented with ActinomycinD (5µM/ml, Sigma-Aldrich, St. Louis, MO) and RNA was extracted at 0, 2, 4 and 6hrs while lymphoblastoid cultures were treated with ActinomycinD (5µM/ml) for 0, 6, 12 and 24hrs. Normalized Ct values were converted to relative mRNA levels, set to 100% for t=0. Decay kinetics were obtained using linear regression analysis and the half life for each examined mRNA was calculated based on the slope and the intercept. The mRNA decay assays were repeated 3 times for each case, results were expressed as the mean ± S.E.M. and statistical significance was determined using Student’s t-test.

### Bioinformatics Analysis

Alignment of the 5’ upstream sequence of the human, mouse and rat *GCH1* genomes was performed using ClustalW (http://www.ebi.ac.uk/Tools/clustalw/). Prediction of RNA secondary structures within the human *CFTR* 5´ UTR mRNA was performed using the mFold program, version 3.2 (http://frontend.bioinfo.rpi.edu/applications/mfold/). The wild type (+142C) and +142T 5’UTR *GCH1* sequences were also analyzed using the UTR site database (utrdb.ba.itb.cnr.it) to detect any RNA protein binding motifs created or destroyed by the +142C>T substitution. Finally, the 5’UTR-Fluc reporter sequences and the human *GCH1* mRNA sequence (NM_000161.2) with either a +142C or a +142T were analyzed on the NCBI ORF Finder (http://www.ncbi.nlm.nih.gov/projects/gorf/) to detect potential uORFs.

## Results

Alignment of the 5’ upstream sequence of the human, mouse and rat *GCH1* genes revealed that among the three point substitutions examined only the +142C is evolutionarily conserved between the human, rat and mouse genome (Figure 1). All of the three 5’ upstream *GCH1* substitutions fall within its 5’UTR (+142C>T; +125C>T; +32T>C; Figure 1) rendering it more likely that any potential effects would involve post-transcriptional mechanisms.

### Effects of the *GCH1* 5’UTR substitution on the luciferase reporter assay

We utilized a previously characterized *GCH1* reporter construct [27] encompassing 452 bps upstream of the transcription start site (+1) and 163 bps corresponding to the distance between the +1 and the translation start site ATG (615GCH1/WT; Figure 2A). Three mutant reporter constructs were generated to mimic the 5’UTR *GCH1* reported substitutions; 615GCH1/+142T, 615GCH1/+125T and 615GCH1/+32C (Figure 2A). Transient transfection assays were performed in a human neuroblastoma SK-N-BE(2)-M17 cell line, a monoamine (MA) and BH4 containing cell line in which the *GCH1* promoter has been reported to be active [28]. Normalized Fluc activity for each of the mutant constructs compared to the wild type (set at 100%) revealed that the 615GCH1/+142T construct caused a reduction in luciferase activity to 32% of the 615GCH1/WT activity (n=4, *P* ≤ 0.005; Figure 2B). Likewise, when the +142T substitution was introduced in the larger 5399 bp reporter construct (5399GCH1/WT and 5399GCH1/+142T, Figure 2C) luciferase activity dropped to 37% of the wild type (n=4, *P* ≤ 0.005; Figure 2D). In contrast, the 615GCH1/+125T, the 615GCH1/+32C and the double mutant 615GCH1/+125T/+32C constructs did not result in a statistically significant reduction in luciferase activity (94%, 89% and 90% of wild type activity, respectively, n=4; Figure 2B). Finally, after generation of the +142T reporter construct, we re-introduced the +142C in the 5399GCH1/+142T or the 615GCH1/+142T constructs and luciferase activity returned to wild type levels (data not shown).

### Effects of the +142C>T *GCH1* substitution on luciferase mRNA

**Figure 3:**
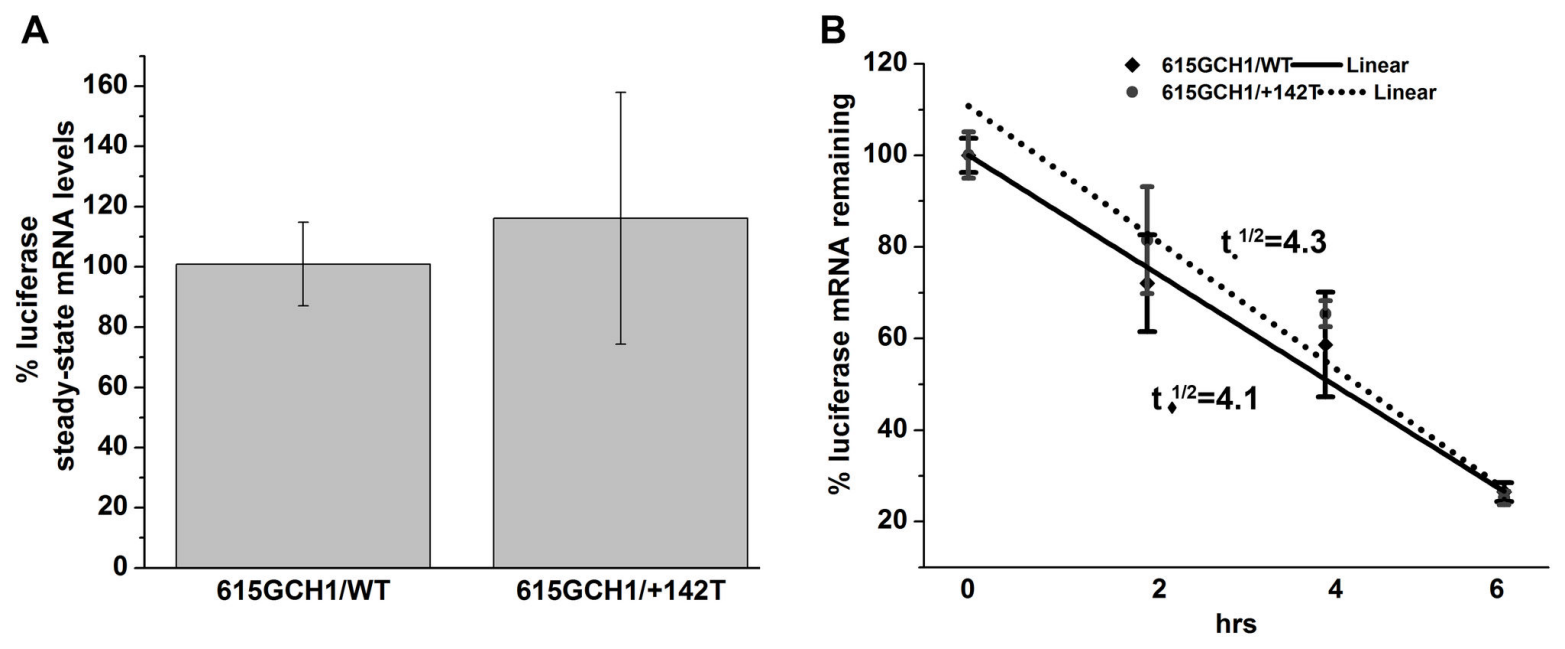
Effects of the +142C>T *GCH1* substitution on the steady-state luciferase mRNA level and its stability. (**A**) Steady-state levels of Fluc/Rluc mRNA in SK-N-BE2-M17 cells transiently transfected with either the 615GCH1/WT or 615GCH1/+142T Fluc and the CMV-Rluc construct. Normalized Fluc Ct values were converted to relative mRNA levels and results are shown as mean ± S.E.M (615GCH1/WT, set at 100%) (n=4). (**B**) SK-N-BE2-M17 cells were transiently transfected as above and treated with ActinomycinD. RNA was collected at 0, 2, 4 and 6 hrs and subjected to qRT-PCR. Normalized Fluc Ct values were converted to relative mRNA levels and results are shown as means ± S.E.M. plotted to a linear regression over time, with time zero set to 100%. Half-lives were calculated based on the slope and intercept; t_♦_^1/2^= 4.1hrs for the 615GCH1/WT construct and t_•_^1/2^= 4.3hrs for the 615GCH1/+142T construct (n=3).

We then analyzed the effects of the +142C>T substitution on luciferase mRNA levels expressed in SK-N-BE(2)-M17 cells transiently transfected with luciferase reporter constructs. QRT-PCRs revealed no statistically significant differences between the relative mRNA levels of the 615GCH1/WT (set to 100%) and the 615GCH1/+142T (113%) reporter constructs (n=4; Figure 3A). To determine if the +142C>T substitution affected mRNA stability, inhibition of transcription was achieved with ActinomycinD and luciferase mRNA half-lives were determined to 4.1 hrs for the 615GCH1/WT and 4.3 hrs for the 615GCH1/+142T transcript. This difference in half-lives was not statistically significant (n=3, Figure 3B).

### Endogenous *GCH1* levels in normal individuals and DRD patients

**Figure 4:**
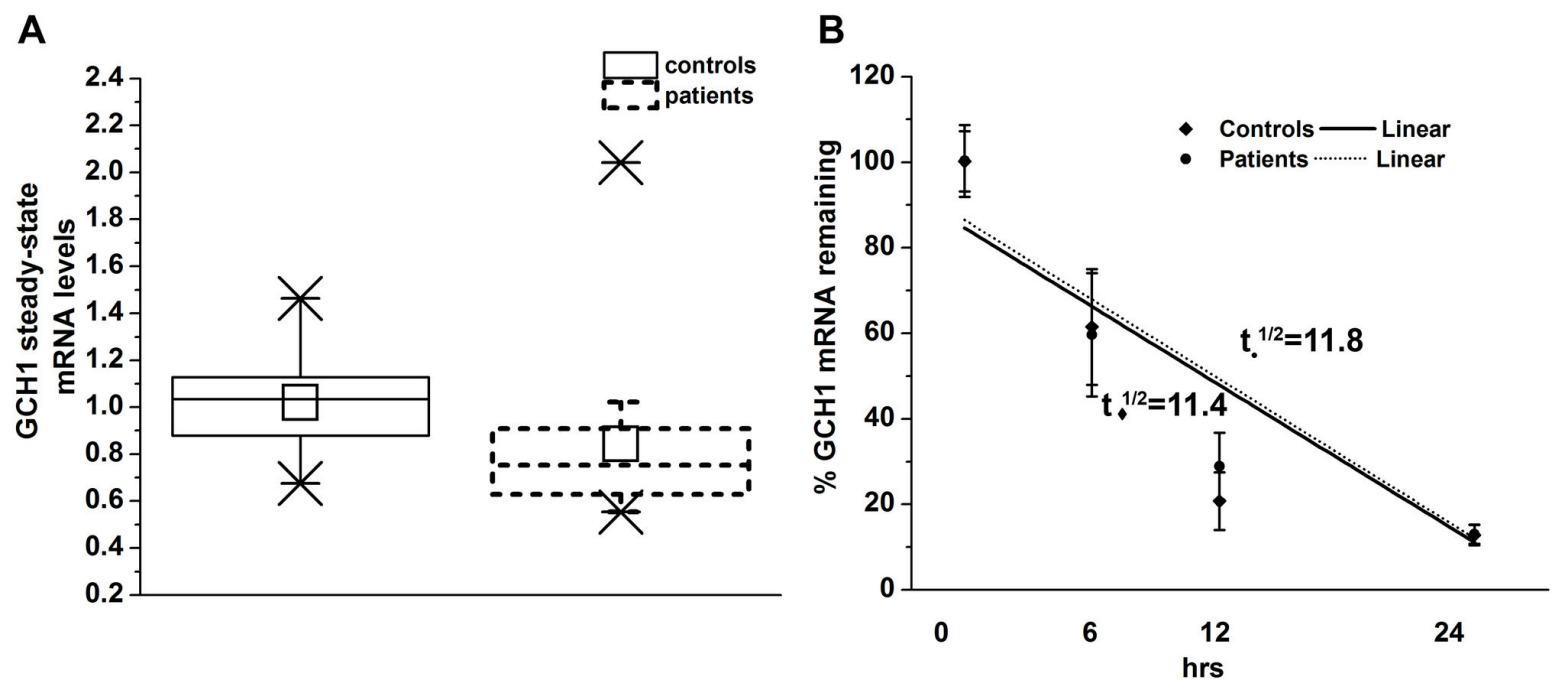
*GCH1* mRNA levels and stability in lymphoblastoid cells from DRD and control subjects. (**A**) Steady-state levels of *GCH1* mRNA in lymphoblastoid cells derived from six DRD subjects and six control subjects. Normalized *GCH1* Ct values were converted to relative mRNA levels. Box plots show mean ± S.E.M. of the control and DRD *GCH1* mRNA levels (n=4). (**B**) Lymphoblastoid cultures from DRD and control subjects were exposed to ActinomycinD, RNA was collected at 0, 6, 12 and 24 hrs and subjected to qRT-PCR. Normalized *GCH1* Ct values were converted to relative mRNA levels and results are shown as means ± S.E.M. plotted to a linear regression over time, with time zero set at 100%. Half-lives were calculated based on the slope and intercept; t_♦_^1/2^=11.4hrs for control and t_•_^1/2^= 11.8hrs for DRD subjects (n=3).

We further investigated the effects of the +142C>T substitution on *in vivo GCH1* mRNA levels by qRT-PCR analysis in lymphoblastoid cultures established from DRD patients and control individuals. While the average relative steady-state levels of *GCH1* mRNA were lower in DRD patients (0.842) compared to controls (set at 1.0), this reduction was not statistically significant (n=4; Figure 4A). Furthermore, when transcription was inhibited by actinomycinD, there was no statistically significant difference in the *GCH1* transcript half-life between DRD and control subjects (11.4 hrs in controls vs 11.8 hrs in patients), (n=3; Figure 4B).

### Bioinformatics analysis

**Figure 5:**
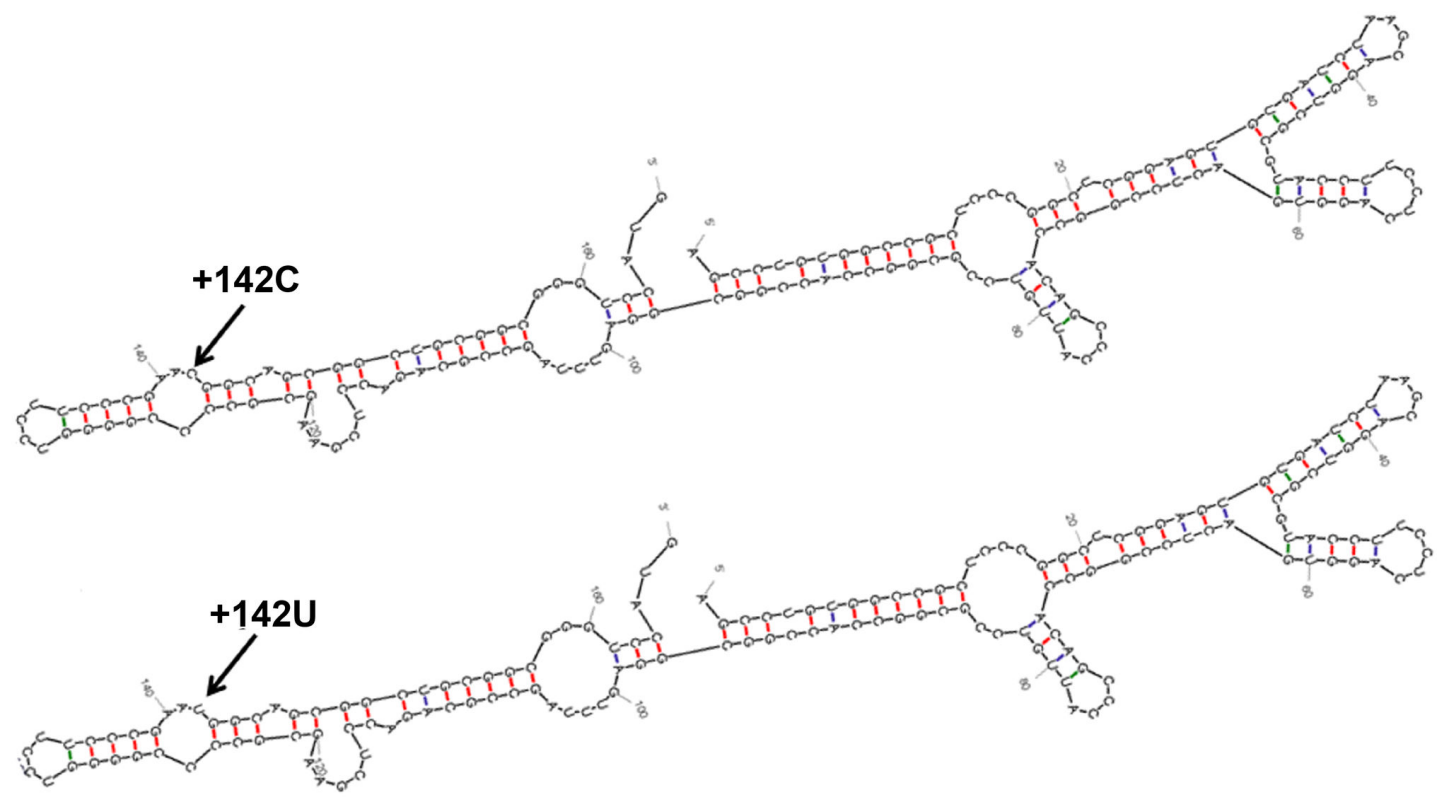
Computer-assisted RNA secondary structures for the +142C and the +142T GCH1 5 upstream region. Mfold analysis predicted same RNA secondary structure regardless of a +142C (up) or a +142T (bottom) within the *GCH1* 5’UTR. The position of the +142C>T substitution is indicated with an arrow.

**Figure 6:**
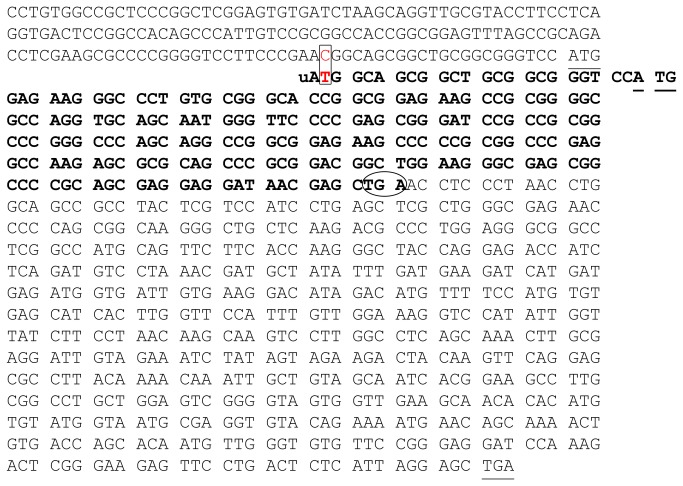
The *GCH1* 5’UTR and Coding region. Normal translation starts at the underlined ATG and ends at the underlined TGA stop codon. The rectangle indicates the point of the +142C>T substitution and the bases in bold indicate the out of frame uORF initiated by the uATG and ending at a TGA triplet (bold, circled) within the coding region of the normal sequence.

RNA secondary structures analysis, performed for the complete 5’UTR *GCH1* region with either a C or a T at +142 predicted no structural differences between them (Figure 5). Since our experimental data did not reveal evidence for transcriptional effects of the +142C>T substitution, we scanned the wild type *GCH1* +142C and the *GCH1* +142T 5’UTRs for RBP motifs and found no differences (data not shown). However, we observed that the +142C>T substitution created a novel upstream ATG codon (ACG>ATG) (Figure 6). Indeed, scanning the 5’UTR-coding *GCH1* sequence with the NCBI ORF Finder software revealed that in presence of a T at +142, an uATG arises and a potential upstream open reading frame (uORF) (sequence in bold; Figure 6), starting 23 nucleotides upstream of the physiological ATG (underlined; Figure 6) and terminating after 219 bases at a STOP codon within an exon of the wild type *GCH1* sequence (TGA circled; Figure 6). Thus, the +142C>T substitution is likely to result in production of a 73 amino acid peptide (MAAAAAGPWRRALCGHRRRSRGAPGAAMGSPSGIRRGPGPAGRRRSPRGPRPRARSPRTAGRASGPAARRITS) which is out of frame with the pATG codon initiating translation of the physiological *GCH1* protein (Figure 6). Note that for the 5’UTR *GCH1*-luciferase constructs used in this study, sequence scanning by the NCBI ORF Finder software showed that the newly generated uORF would encode an out of frame of 14aa truncated peptide (analysis non shown).

## Discussion

In eukaryotes, regulation of protein synthesis is a finely-tuned process involving multiple steps of transcription, posttranscriptional processing and modification, mRNA stability, translation initiation and posttranslational modification. Human disease can result from single nucleotide polymorphisms that affect any step of this finely tuned regulatory system. In particular for diseases linked to non-coding substitutions, functional assays are required to establish a link between the substitution and the disease phenotype. In DRD, three different sequence substitutions have been identified within the 5’ untranslated region of the *GCH1* gene [16–18]. We demonstrated here that the +125C>T and the +32T>C substitutions reported together in a single DRD patient had negligible effects on luciferase activity when tested either individually or combined into a single reporter construct. The presence of these substitutions has been examined in only 3 neurologically normal subjects, who did not harbor either or both substitutions [17]. In contrast, the +142C>T substitution which segregates with affected status in ten members of a single DRD family, spanning three generations but not found in a total of 543 control alleles [18], had a strong negative effect, leading to a 68% reduction in luciferase activity. The effect persisted even when the +142T substitution was introduced into the larger 5399GCH1 reporter construct having a greater degree of endogenous transcriptional regulation. To ensure that no random mutations within the luciferase vector backbone affected our results, we re-introduced the wild type +142C back onto the mutant +142T construct and luciferase activity returned to wild type levels. To our knowledge, no gene dosages studies of the +142C>T substitution carriers has been conducted.

We next investigated if the +142T-triggered reduction in luciferase activity was mediated by a reduction in luciferase mRNA levels. We found that the +142T had no significant effect on luciferase mRNA abundance. Furthermore, RNA decay kinetics demonstrated a similar mRNA half-life for both the +142C and the +142T 5’UTR luciferase reporter constructs. Similarly, there was no statistically significant difference in endogenous steady-state *GCH1* mRNA levels or its stability in lymphoblastoid cells derived from DRD and control subjects. Cerebrospinal fluid from these patients was not available to assess the BH_4_ level but previous studies support that altered *GCH1* mRNA levels can be detected in mononuclear blood cells from DRD patients with coding *GCH1* mutations [29]. In agreement with our experimental data, bioinformatics analysis predicted the same RNA structure in the presence of a +142C or a +142T within the *GCH1* 5’UTR. Taken together these results support that the +142C>T *GCH1* 5’UTR substitution alters efficiency of translation without affecting RNA levels or RNA stability/structure.

Scanning of the wild type +142C and the +142T *GCH1* 5´ UTR sequences predicted no differences in RBP sites. Thus, it is unlikely that differences between RBP association with the wild type and the 142T 5’UTR sequences are the cause of the observed changes in translation. An alternate level of translational regulation involves the creation of uAUGs and uORFs [25]. Genes can be devoid of uAUGs or bear a single or several uAUGs which mediate complete or partial inhibition of the physiological AUG (pAUG) [22,25,30]. Our *in silico* analysis revealed that the wild type 5’UTR *GCH1* region lacks of any uAUG. However, the +142T substitution introduces a single uAUG and a potential uORF encoding a truncated, out of frame, peptide. The +142T substitution results in reduced luciferase activity indicating that both the truncated and full length luciferase protein are generated, with the truncated peptide competing for efficiency of translation against the full length peptide.

These results do not provide direct evidence of the effects of the +142T substitution on the *GCH1* levels in DRD patients. However, similar to the luciferase constructs, the +142T may generate an uORF that encodes a 73 aa out of frame peptide that competes with the translation of wild type *GCH1* initiated by the pAUG. A model in which a mixture of wild type and truncated peptides are generated in a competitive manner due to creation of novel uAUGs has been reported in human diseases ranging from a predisposition to cancer [31] to bronchiectasis [32] and bipolar depression [33]. Our study expands this list to include dystonia by revealing that the +142C>T *GCH1* 5’UTR substitution detected in DRD patients is a functional mutation that reduces translational efficiency and likely leads to reduced wild type *GCH1* protein levels, underlying manifestation of DRD.
