# Correction: Dopa-Responsive Dystonia: Functional Analysis of Single Nucleotide Substitutions within the 5’ Untranslated *GCH1* Region

**DOI:** 10.1371/annotation/4ace7171-c9d7-490b-a7d6-b49be4094281

**Published:** 2013-10-23

**Authors:** Ioanna A. Armata, Leonora Balaj, John K. Kuster, Xuan Zhang, Shelun Tsai, Andreas A. Armatas, Trisha J. Multhaupt-Buell, Roy Soberman, Xandra O. Breakefield, Hiroshi Ichinose, Nutan Sharma

A funding organization and funding program were incorrectly omitted from the Funding Statement. The Funding Statement should read: "This work was supported by NIH/NINDS 5P50NS037409 (XOB and NS) and by the Parkinson's and Movement Disorder's Foundation (PMDF 2011)." 

